# Pretreatment quality of life in patients with rectal cancer is associated with intrusive thoughts and sense of coherence

**DOI:** 10.1007/s00384-017-2900-y

**Published:** 2017-09-14

**Authors:** Dan Asplund, Thue Bisgaard, David Bock, Jakob Burcharth, Elisabeth González, Eva Haglind, Yanislav Kolev, Peter Matthiessen, Carina Rosander, Jacob Rosenberg, Kenneth Smedh, Marina Åkerblom Sörensson, Eva Angenete

**Affiliations:** 1 Department of Surgery, Institute of Clinical Sciences, Sahlgrenska Academy, University of Gothenburg, Scandinavian Surgical Outcomes Research Group (SSORG), Sahlgrenska University Hospital/Östra, 416 85 Gothenburg, Sweden; 2 Department of Surgery, Hvidovre Hospital, University of Copenhagen, Copenhagen, Denmark; 3 Department of Surgery, Herlev Hospital, University of Copenhagen, Copenhagen, Denmark; 4 Department of Surgery, NU Hospital group, Trollhättan, Sweden; 5 Department of Surgery, Faculty of Medicine and Health, Örebro University, Örebro, Sweden; 6 Department of Surgery, Västmanland’s Hospital Västerås, 721 89 Västerås, Sweden; 7 Department of Surgery, Central Hospital of Karlstad, Karlstad, Sweden

**Keywords:** Cancer, Oncology, Rectal cancer, Quality of life, Intrusive thoughts, Sense of coherence, Clinical trial

## Abstract

**Purpose:**

Quality of life may predict survival. In addition to clinical variables, it may be influenced by psychological factors, some of which may be accessible for intervention. The primary objective of this study was to investigate the association of intrusive thoughts and the patients’ sense of coherence with pretreatment quality of life in patients with newly diagnosed rectal cancer.

**Methods:**

Patients were prospectively included in 16 hospitals in Sweden and Denmark. They answered an extensive questionnaire after receiving their treatment plan. Clinical data were retrieved from national quality registries for rectal cancer.

**Results:**

Of 1248 included patients, a total of 1085 were evaluable. Pretreatment global health-related and overall quality of life was lower in patients planned for palliative compared with curative treatment (median 53 vs. 80 on the EuroQoL visual analogue scale, *p* < 0.001 and odds ratio 0.56, 95% confidence interval 0.36–0.88, respectively). Quality of life was associated with intrusive thoughts (odds ratio 0.33, 95% confidence interval 0.24–0.45) and sense of coherence (odds ratio 0.44, 95% confidence interval 0.37–0.52) irrespective of the treatment plan.

**Conclusions:**

Pretreatment quality of life was influenced by the intent of treatment as well as by intrusive thoughts and the patients’ sense of coherence. Interventions could modify these psychological factors, and future studies should focus on initiatives to improve quality of life for this group of patients.

## Introduction

Rectal cancer is potentially life threatening. Curative treatment is attempted in about 80% while remaining patients receive palliative treatment [17]. Survival has improved in recent years, and consequently, quality of life (QoL) has received increasing attention [1]. Interestingly, several studies have found QoL to be predictive of survival in patients with cancer [9, 25, 31]. Furthermore, one study has demonstrated the prognostic significance of pretreatment QoL for survival in primary rectal cancer [8]. Several factors may influence pretreatment QoL in patients with newly diagnosed rectal cancer, including the cancer disease per se [12]. QoL could be influenced by the treatment plan, i.e., curative or palliative intent and the type of surgical procedure planned. Several studies have found QoL to be associated with psychological and personality trait variables independent of clinical factors [13, 27, 32, 33]. Cancer-related intrusive thoughts, i.e., unintentional recurrent or distressing thoughts about cancer, are among the more prominent stress-related symptoms experienced by patients with cancer [15, 30]. An association has been reported between intrusive thoughts and QoL in patients with prostate cancer [37, 41], but this has not been studied in patients with rectal cancer. Another psychological variable that may be associated with QoL is sense of coherence (SOC), which reflects a person’s view on life and capacity to respond to stressful situations [2, 10]. Sense of coherence was developed by Aaron Antonovsky in the 1970s [2] and may be regarded as a test of personality traits or coping disposition. It mirrors the extent to which we perceive life as comprehensible, manageable, and meaningful [24]. An association between sense of coherence and quality of life has been reported in several studies [10].

We hypothesized that pretreatment quality of life is influenced by psychological factors irrespective of clinical variables like intent of treatment. The primary objective of the present analysis was to investigate the association of intrusive thoughts and the patients’ sense of coherence with pretreatment quality of life in patients with newly diagnosed rectal cancer.

## Methods

### Study design

The QoLiRECT study is an ongoing prospective observational multicenter study of quality of life and functional outcome in patients with rectal cancer [3]. All patients aged above 18 years with a biopsy-confirmed rectal adenocarcinoma were eligible for inclusion, regardless of tumor stage or planned treatment. The only exclusion criterion was inability to understand and respond to the questionnaire due to language difficulties or to cognitive failure. Inclusion began in February 2012 and was terminated in September 2015 and took place when the diagnostic work-up was complete and the patient had been presented with a treatment plan, but before treatment had started. Informed consent was obtained from all included patients. Patients will be followed for 5 years with data collection at baseline as well as 1, 2, and 5 years after inclusion. The present analysis is concerned with baseline pretreatment data only.

### Data collection

At inclusion, patients completed a comprehensive pretreatment questionnaire which included questions on overall QoL and global health-related QoL, intrusive thoughts, and sense of coherence. Patients were contacted by the study secretariat by phone within a few days after inclusion and received the questionnaire by mail. In hospitals with short lead times to start of treatment, the questionnaire was handed to the patient at inclusion and was returned to the study secretariat by prepaid envelope. Questionnaires were returned to the secretariat before treatment was initiated.

At inclusion, patients were registered as candidates for either curative or palliative treatment. For curative patients, it was indicated whether the treatment plan included an abdominoperineal excision (APE) or not. This information was registered in a database at the study secretariat along with name, address, and other basic personal data needed for logistic reasons. All other clinical data, including pretreatment staging, were collected from the national quality registries (the Swedish ColoRectal Cancer Registry [26] and the national database of the Danish Colorectal Cancer Group [14]) or hospital administrative systems.

### Questionnaire development

The development of the study specific questionnaire has been described in detail elsewhere [3]. The questionnaire was developed according to a well-established method [18, 36] including semi-structured interviews with patients followed by construction of questions and subsequent content and face-to-face validation. It was forward and backward translated into Danish and then face-to-face validated once more as previously described [3]. Parts of the questionnaire have been used previously in a cross-sectional Swedish national study of patients with rectal cancer treated with abdominoperineal excision [4]. The questionnaire included novel questions specific for rectal cancer as well as questions previously used in patients with urological and gynecological cancer [6, 36, 38]. Also included was the EQ-5D-3L [7] as well as questions on cancer-related intrusive thoughts [37] and the 29-item Sense of Coherence scale (SOC-29) [2], a validated and cross-culturally applicable instrument to measure sense of coherence [11].

### Outcome measures

Overall QoL was assessed by the question “How would you describe your quality of life during the past month?” with a seven-point Likert scale response format anchored by zero (“No quality of life”) and six (“Best possible quality of life”). Global health-related QoL was assessed by the EQ-5D-3L visual analogue scale [7, 29], anchored by 0 (worst imaginable health state) and 100 (the best imaginable health state).

### Explanatory variables

Cancer-related intrusive thoughts were assessed by two questions pertaining to the frequency and severity of intrusions: “How often during the past month have you had negative thoughts about your rectal cancer, suddenly and unintentionally?”, assessed by an ordinal scale with seven levels ranging from “Never” to “More than 3 times per day or all the time,” and “How intrusive have you experienced the sudden negative thoughts about your rectal cancer?”, with response options “Not at all intrusive,” “A little bit intrusive,” “Moderately intrusive,” and “Very intrusive” as well as “Not applicable.” These variables were dichotomized for the analysis as explained in the corresponding tables.

Sense of coherence [2] was evaluated by SOC-29, which consists of 29 items and three domains: 11 comprehensibility items (e.g., “Do you have the feeling that you are in an unfamiliar situation and don’t know what to do?”), 10 manageability items (e.g., “How often do you have feelings that you are not sure you can keep under control?”), and eight meaningfulness items (e.g., “Do you have the feeling that you don’t really care about what goes on around you?”). Each item is assessed by a seven-point Likert scale, and items are summated into a total score that ranges from 29 to 203.

The third explanatory variable was the treatment plan which was either curative or palliative. Patients planned for curative treatment were further subgrouped as candidates for APE with a permanent stoma, or other curative procedures.

### Possible confounders

Comorbidity was characterized by a number of health conditions, including joint disorders, cardiovascular, neurologic, pulmonary, renal, bowel and psychological conditions as well as diabetes and chronic pain, and defined as the presence of at least one of these conditions. Depression was treated as a separate confounder and was evaluated by a validated single-item question [34]. Age, sex, marital status, education, occupation, and time from diagnosis were also adjusted for in the statistical analysis as well as whether the patient considered him or herself well informed about the cancer and the planned treatment.

### External validity

**Fig. 1:**
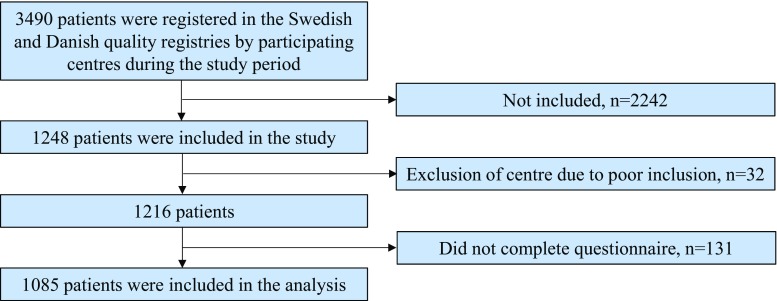
Flowchart of patients. Non-included patients (*n* = 2242) did not meet the inclusion criteria or were missed to inclusion

Selection bias was evaluated by comparing included and non-included patients at participating departments of surgery during the inclusion period (Fig. 1). Data were retrieved on sex, age, ASA (American Society of Anesthesiologists) grade, clinical TNM status, and tumor height from the national quality registries [14, 26] and hospital administrative systems.

### Population normative data

**Fig. 2:**
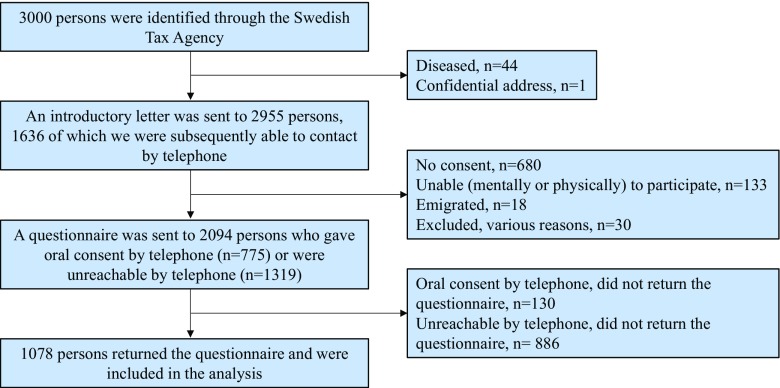
Flowchart of persons included in the reference population sample

A representative cohort of 3000 Swedish men and women born 1924–1983 was randomly selected from the general population through the Swedish Tax Agency and contacted by letter and telephone (Fig. 2). A total of 1078 persons (median age 63 years, range 31–90; 53% female) accepted to participate and completed a questionnaire similar to the study questionnaire. This allowed for comparison of results to the population norm.

### Statistical analysis

A detailed statistical plan was developed prior to data analysis. A proportional odds model [22] was used to explore the association of overall QoL (“How would you describe your quality of life during the past month?”) and the explanatory variables. Results were presented as odds ratios with 95% confidence intervals. A confidence interval covering one was considered non-significant. Adjusted analyses were performed with the potential confounders included as covariates. Although overall quality of life was dichotomized in Table 5 as done previously [36, 37], the odds ratios are based on the original (continuous) seven-point Likert scale. The impact of the explanatory variables on global health-related QoL (EQ-5D-3L visual analogue scale) was analyzed using Mann-Whitney test and Spearman’s rank correlation.

In the calculation of the SOC-29 total sum score, missing values in 25% or less of the items were replaced by the median score for the remaining answered items. If more than 25% of the items had missing values, the total score was regarded as missing.

Statistical analyses were performed using SPSS 21.0 (IBM SPSS Inc. Armonk, NY, USA) and SAS v. 9 (SAS institute).

Appropriate permission was obtained from the Danish Data Protection Agency (HEH.750.89-21; HGH-2016-016) and the Regional Ethical Review Board in Sweden (EPN 595-11) and Denmark (H-3-2012-FSP26). Permissions to use SOC-29 and EQ-5D-3L were also obtained. The study was registered at ClinicalTrials.gov (NCT01477229).

## Results

Between February 2012 and September 2015, 16 departments of surgery in Sweden and Denmark prospectively included 1248 patients with newly diagnosed rectal cancer, of which 1085 patients were available for analysis (Fig. 1). There were no significant differences regarding patient demographics between patients to be treated with curative or palliative intent (Table 1) or any clinically relevant differences regarding age, sex, or tumor height between these groups (Table 2), but as expected, the tumor stage differed. The distribution of sex, age, ASA grade, and clinical tumor stage differed significantly between patients included in the study and non-included patients (Table 3).

**Table 1:** Demography of the study group

	All, *n* = 1085	Curative, *n* = 1012	Palliative, *n* = 73	*p* value	Missing
Marital status (%)				n.s.	11
In a relationship	796 (74)	747 (75)	49 (68)		
Not in a relationship	278 (26)	255 (25)	23 (32)		
Education (%)				n.s.	19
University	198 (19)	189 (19)	9 (13)		
No university	868 (81)	806 (81)	62 (87)		
Occupation (%)				n.s.	17
Working	311 (29)	292 (29)	19 (26)		
Retired	692 (65)	645 (65)	47 (65)		
Unemployed	14 (1)	14 (1)	0 (0)		
Sick leave	51 (5)	45 (5)	6 (8)		
Comorbidity (%)				n.s.	84
Yes	661 (61)	610 (61)	51 (70)		
No	412 (38)	390 (39)	22 (30)		
Depression (%)				n.s.	9
Yes/do not know	181 (17)	165 (16)	16 (22)		
No (%)	895 (83)	839 (84)	56 (78)		
Time since diagnosis (%)				n.s.	24
0–1 week	165 (16)	152 (15)	13 (18)		
1–2 weeks	192 (18)	180 (18)	12 (17)		
2–4 weeks	405 (38)	377 (38)	28 (38)		
> 4 weeks	299 (28)	279 (28)	20 (27)		

n.s. = *p* value > 0.05

**Table 2:** Clinical characteristics of the study group

	All	Curative			Palliative	*p* value^c^
		All	Not APE	APE		
Number of patients	1085	1012	740	272	73	
Sex (%)						n.s.
Female	490 (45)	460 (45)	331 (45)	129 (47)	30 (41)	
Male	595 (55)	552 (55)	409 (55)	143 (53)	43 (59)	
Age, median (range)	69 (25–100)	69 (25–100)	68 (25–100)	70 (38–91)	70 (35–96)	< 0.05
Tumor stage, cTNM^a^ (%)						
T1–T2	269 (25)	268 (26)	199 (27)	69 (25)	1 (1)	< 0.001
T3	559 (51)	525 (52)	399 (54)	126 (46)	34 (47)	
T4	158 (15)	128 (13)	74 (10)	54 (20)	30 (41)	
TX	99 (9)	91 (9)	68 (9)	23 (9)	8 (11)	
N0	426 (39)	416 (41)	306 (41)	110 (40)	10 (14)	< 0.001
N1–N2	532 (49)	484 (48)	352 (48)	132 (49)	48 (66)	
NX	127 (12)	112 (11)	82 (11)	30 (11)	15 (20)	
M0	894 (82)	877 (87)	639 (87)	238 (87)	17 (23)	< 0.001
M1	124 (12)	77 (7)	61 (8)	16 (6)	47 (64)	
MX	67 (6)	58 (6)	40 (5)	18 (7)	9 (12)	
Tumor height^b^, median (range)	8 (0–15; IQR 6)	8 (0–15; IQR 6)	10 (0–15; IQR 4)	4 (0–15; IQR 3)	9 (2–15; IQR 7)	n.s.

n.s. = *p* value > 0.05

^a^Clinical TNM stage, based on radiology

^b^Measured in centimeters from the anal verge on retraction of a rigid rectoscope in the left lateral position. Data missing in 64 patients

^c^Curative vs. palliative patients

**Table 3:** Comparison between the study group and the non-included patients

	Study group	Non-included^a^	*p* value
Number of patients	1085	2242	
Sex (%)			< 0.001
Female	490 (45)	882 (39)	
Male	595 (55)	1360 (61)	
Age, median (range)	69 (25–100)	70 (23–100)	< 0.001
ASA grade^b^ (%)			< 0.001
1	230 (24.5)	394 (22)	
2	560 (60)	982 (54)	
3	142 (15)	404 (22)	
4	5 (0.5)	28 (2)	
Tumor stage, cTNM^c^ (%)			
T1–T2	269 (25)	484 (22)	< 0.001
T3	559 (51)	936 (42)	
T4	158 (15)	475 (21)	
TX	99 (9)	347 (15)	
N0	426 (39)	420 (19)	< 0.001
N1–N2	532 (49)	742 (33)	
NX	127 (12)	1080 (48)	
M0	894 (82)	1749 (78)	< 0.001
M1	124 (12)	408 (18)	
MX	67 (6)	85 (4)	
Tumor height^d^, median (range)	8 (0–15; IQR 6)	8 (0–15; IQR 6)	n.s.

n.s. = *p* value > 0.05

^a^Patients treated at participating hospitals during the inclusion period but not included in the study

^b^ASA (American Society of Anesthesiologists) grade is missing in patients scheduled for palliative treatment

^c^Clinical TNM stage, based on radiology

^d^Measured in centimeters from the anal verge on retraction of a rigid rectoscope in the left lateral position. Data were missing in 64 patients in the study group and in 55 non-included patients

In the study group, intrusive thoughts were frequent among all patients without any significant differences between patients planned for curative and palliative treatment (Table 4). Sense of coherence differed slightly between groups (Table 4).

**Table 4:** Distribution of psychological explanatory variables among patients planned for curative and palliative treatment

	All, *n* = 1085	Curative, *n* = 1012	Palliative, *n* = 73	*p* value	Missing
Sense of coherence, mean (SD; range)	158 (20; 85–203)	159 (20; 85–203)	153 (22; 98–196)	< 0.05	29
Intrusive thoughts (%)				n.s.	16
No	184 (17)	170 (17)	14 (19)		
Yes	885 (83)	826 (83)	59 (81)		
Frequency (%)				n.s.	16
Low^a^	271 (31)	254 (31)	17 (29)		
High^a^	614 (69)	572 (69)	42 (71)		
Intrusiveness (%)				n.s.	62
Low^b^	551 (67)	516 (67)	35 (65)		
High^b^	272 (33)	253 (33)	19 (35)		

n.s. = *p* value > 0.05

^a^Less than once per week/at least once per week

^b^Less than moderately intrusive/moderately or very intrusive

Patients planned for palliative treatment assessed their overall QoL and global health-related QoL as lower than patients with a curative treatment plan (Table 5). Patients with a curative treatment plan had similar global health-related QoL as a general population, but significantly lower overall QoL (data not shown). Patients planned for APE reported lower overall QoL, but not lower global health-related QoL, compared with patients planned for other types of curative treatment (Table 5). The experience of intrusive thoughts was associated with lower overall QoL and global health-related QoL. The higher the frequency and perceived intrusiveness of these thoughts, the greater was the impact on QoL (Table 5). A 25-unit increase in the total score of SOC-29 was associated with better overall QoL (odds ratio 0.44, 95% confidence interval 0.37–0.52), and sense of coherence was positively correlated with global health-related QoL (Spearman correlation 0.44; *p* < 0.001).

**Table 5:**
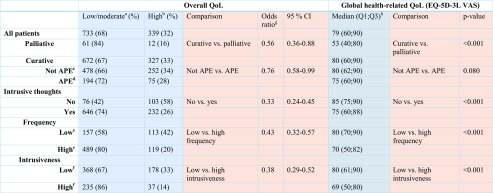
Association of planned treatment and intrusive thoughts with overall and global health-related QoL

Overall QoL is presented here as a dichotomized outcome (blue left columns), but the odds ratios for the comparisons between subgroups (pink left columns) are based on the original seven-point Likert scale

^a^Zero to four on a seven-point Likert scale

^b^Five to six on a seven-point Likert scale

^c^Patients operated by other curative procedures than abdominoperineal excision

^d^Patients operated by abdominoperineal excision

^e^Less than once per week/at least once per week

^f^Less than moderately intrusive/moderately or very intrusive

^g^Odds ratio for a lower overall QoL, adjusted for age, sex, comorbidity, depression, occupation, education, marital status, sense of coherence, time from diagnosis, and whether the patient regarded him or herself well informed about the cancer and planned treatment

^h^Data missing in 87 patients

A total of 97% of the patients planned for curative treatment considered themselves well informed about their diagnosis and treatment plan vs. 85% of the patients planned for palliative treatment, (*p* < 0.001).

## Discussion and conclusions

In the present study, we found that QoL was lower in patients planned for palliative compared with curative treatment. Patients with potentially curable disease had a global health-related QoL comparable to that of a general population, but a significantly lower overall QoL. It is important to note that low QoL was associated with the experience of intrusive thoughts, a possibly modifiable factor. QoL was also associated with sense of coherence, a personal trait factor.

The physical effects of the disease burden as well as the psychological reaction to the palliative situation may partly explain the low QoL in patients planned for palliative treatment. One such reaction is intrusive thoughts which may be modified by expressive writing, cognitive behavioral therapy, and pharmacological interventions [19, 23]. Although previously thought to be intrinsic and non-modifiable, recent preliminary data have indicated that sense of coherence may indeed be strengthened by psychotherapy [16].

There were no clinically relevant differences of overall QoL or global health-related QoL between patients planned for curative treatment with APE vs. other curative procedures [29]. In view of the fact that 97% of the curative patients felt well informed about their treatment, this indicates that patients received adequate supportive care when presented with their treatment plan, including patients scheduled for a permanent stoma. As recently reported, Swedish patients’ QoL was not limited by the presence of a permanent stoma following APE for rectal cancer [21].

A lower percentage of patients planned for palliative treatment considered themselves well informed about their diagnosis and treatment plan compared with patients planned for curative treatment. This indicates a need for improved communication of palliative treatment, perhaps in a more structured manner [28, 40]. In this context, better support for the surgeon may ultimately improve patient outcome [5].

QoL was assessed by two global single questions pertaining to “overall” and “health-related” QoL, respectively. While the latter question concerns the patients’ self-rated health state, the question on overall QoL relates also to other aspects of life that may affect QoL. In view of this, it is interesting that patients with a potentially curable disease differed from a normal population with regard to overall but not global health-related QoL, meaning that their “health state” was not impaired by the cancer but their general perception of QoL was.

Strengths of this study include the large patient cohort and the inclusion of patients regardless of tumor stage. The participation of university as well as county hospitals increases the generalizability of results. Study hypotheses and a detailed statistical analysis plan were decided on prior to data analyses. The questionnaire was developed in a systematic way using prevalidated questions as well as newly constructed disease-specific questions validated according to accepted principles [3, 6, 18, 36, 37]. The patients completed the questionnaire at home and returned it to the coordinating center and not to the treating hospital which may minimize bias [20].

Missing data were generally low. However, for global health-related QoL, missing and/or invalid values were more frequent, which we found to be partly explained by difficulties for patients to correctly interpret instructions on how to fill out their response on the EQ-5D-3L visual analogue scale. This may potentially bias the results, but the direction and significance of this bias are unclear.

Women were overrepresented among included compared to non-included patients, but the sex distribution in the study group was comparable to that reported for all new cases of colorectal cancer in Europe 2012 [39]. Median age differed significantly between included and non-included patients, but the 1-year difference is hardly of any clinical relevance in relation to QoL. Included patients had a significantly lower ASA grade, indicating that they were somewhat healthier than non-included patients. Clinical tumor stage was significantly lower in the study group. The differences in ASA grade and clinical tumor stage between included and non-included patients must be considered in relation to the generalizability of results, although the clinical relevance of the rather small differences is difficult to determine.

A good understanding of Swedish or Danish was required for participation, and thus, the results are not valid for patients with little or no knowledge of these languages. Another limitation of the study is that we did not have detailed information on how the treatment plan was communicated to patients. However, many participating departments of surgery and caregivers may reduce the influence of differences in communication and may reflect real life clinical practice at the time.

Although efforts were made to recruit palliative patients to the study, they seem somewhat underrepresented. However, the classification of patients as curative or palliative was done prospectively at the time of inclusion. As some patients initially scheduled for curative treatment were not operated, the number of missed palliative patients may actually not be as large as it looks. If a selection bias is present with regard to patients planned for palliative treatment, it may lead to an overestimation of QoL in this group.

Our results indicate that psychological factors need to be considered as confounders in clinical studies that aim to describe quality of life. Furthermore, reducing intrusive thoughts by way of a simple intervention like expressive writing [23] may offer patients relief and possibly improve quality of life in the face of a rectal cancer diagnosis.

The reported association of QoL and survival in patients with cancer in general [9, 25, 31], and of pretreatment QoL and survival in primary rectal cancer in particular [8], underpins the importance of our results. Furthermore, a survival benefit of psychosocial intervention in patients with cancer has been suggested [35]. However, whether efforts to improve pretreatment QoL will translate into a better survival in patients with rectal cancer remains to be demonstrated.

We conclude that the QoL of Scandinavian patients who had just received the treatment plan for their newly diagnosed rectal cancer may be influenced by the presence of intrusive thoughts and the patients’ sense of coherence as well as of the intent of treatment. Future studies should evaluate the effect of interventions aimed to improve QoL.
